# Effect of a 12-week home-based exercise training program on aerobic capacity, muscle mass, liver and spleen stiffness, and quality of life in cirrhotic patients: a randomized controlled clinical trial

**DOI:** 10.1186/s12876-022-02147-7

**Published:** 2022-02-14

**Authors:** Pavapol Sirisunhirun, Wimolrak Bandidniyamanon, Yonworanat Jrerattakon, Kobkun Muangsomboon, Pornpoj Pramyothin, Supot Nimanong, Tawesak Tanwandee, Phunchai Charatcharoenwitthaya, Siwaporn Chainuvati, Watcharasak Chotiyaputta

**Affiliations:** 1 Division of Gastroenterology, Department of Medicine, Faculty of Medicine Siriraj Hospital, Mahidol University, 2 Wanglang Road, Bangkoknoi, Bangkok, 10700 Thailand; 2 Division of Health Promotion, Faculty of Medicine Siriraj Hospital, Mahidol University, Bangkok, Thailand; 3 Department of Radiology, Faculty of Medicine Siriraj Hospital, Mahidol University, Bangkok, Thailand; 4 Division of Nutrition, Department of Medicine, Faculty of Medicine Siriraj Hospital, Mahidol University, Bangkok, Thailand

**Keywords:** Home-based exercise, Cirrhosis, Aerobic capacity, Sarcopenia, Health-related quality of life

## Abstract

**Background:**

Physical inactivity and sarcopenia are two important predictors associated with increased morbidity and mortality in patients with cirrhosis. At present, the benefit of a home-based exercise training program is not well established in cirrhotic patients. The main objective of this study was to evaluate the effect of a 12-week home-based exercise training program on aerobic capacity in cirrhotic patients.

**Methods:**

This is a randomized controlled study. Patients with compensated cirrhosis were randomized by a block of 4 with concealed allocation to the home-based exercise training (n = 20) or control (n = 20). Both groups received protein supplementation (9 g/day) for 12 weeks. The home-based exercise training program included several aerobic/isotonic moderate-intensity continuous training exercises for 40 min per session, at least four times a week, with a total duration of 12 weeks. The heart rate was continuously monitored using a Garmin® watch. In the control group, patients received exercise instruction without active encouragement and continuous monitoring. The primary outcome was a change in the 6-min walk test from baseline. Secondary outcomes were the difference in thigh muscle thickness, liver stiffness, spleen stiffness, and quality of life.

**Results:**

A total of 40 patients were enrolled prospectively. The mean age was 56.3 ± 7.8 years, with a male predominance of 65%. The mean body mass index was 25.23 ± 3.0 kg/m^2^, and all were Child–Pugh A. Chronic hepatitis B or C was the primary cause of cirrhosis. The baseline values were a 6-min walk test of 475 ± 70 m, liver stiffness of 15.3 ± 9.3 kPa, spleen stiffness of 29.8 ± 21.7 kPa, and thigh muscle thickness (average compression index) of 0.64 ± 0.2 cm/m^2^. All baseline characteristics between the two groups were not different except the mean muscle mass which was significantly higher in the home-based exercise training group (*p* = 0.03, 95% CI 0.01 to 0.17). At the end of the study, no significant difference in the 6-min walk test was observed (*p* = 0.36, 95% CI −15.5 to 41.7). Liver stiffness measurement significantly improved in both groups, but no significant difference between groups was demonstrated (*p* = 0.77, 95% CI −1.3 to 1.8). Thigh muscle thickness was not different between groups. The fatigue domain of the quality of life index was significantly improved in the home-based exercise training group compared with the control group (*p* = 0.05, 95% CI 0.00 to 0.67). No adverse events occurred in a home-based exercise training program.

**Conclusions:**

A 12-week moderate-intensity home-based exercise training program in compensated cirrhotic patients significantly improved the fatigue domain of the quality of life index without an increase in adverse events. However, no benefit in terms of aerobic capacity, thigh muscle mass, liver stiffness, and spleen stiffness was demonstrated.

*Trial registration*: Thai Clinical Trials Registry number TCTR20190926002, 26/09/2019 (Retrospectively registered).

**Supplementary Information:**

The online version contains supplementary material available at 10.1186/s12876-022-02147-7.

## Background

Exercise plays an essential role in patients with various chronic diseases. Exercise is recommended in many clinical practice guidelines, including the care of patients with chronic heart failure, chronic lung disease, and post-solid organ transplantation [1–3]. There is well-established evidence showing that exercise improves frailty and sarcopenia in patients with other chronic conditions. However, no current guidelines recommend exercise for patients with chronic liver disease. Although the results of previous studies indicated the benefit of exercise in this subgroup of patients, many clinicians hesitate to suggest exercise to cirrhotic patients due to the concerns regarding portal hypertension-related complications, especially in patients with decompensated cirrhosis[4, 5]. Moreover, most patients with chronic liver disease tend to be physically inactive, have a sedentary lifestyle, and be further complicated by malnourishment. These tendencies often result in frailty and sarcopenia in cirrhotic patients, which are significant predictors of increased morbidity and mortality.

Several studies have investigated the benefit and safety of exercise in cirrhotic patients [6–11]. The reported advantages of exercise were improved aerobic capacity, increased muscle mass, and improved QoL. Exercise in cirrhotic patients has also been shown to decrease hepatic venous pressure gradient (HVPG) [10, 11]. The mechanism of decreased HVPG may be via a reduction in hepatic vascular resistance, which differs from the effect of non-specific beta-blocker. The main disadvantages of supervised hospital-based exercise employed in most studies were generalizability, practicality, and sustainability, especially after the end of the study period.

Home-based exercise training (HoBET) is an alternative intervention for cirrhotic patients that can be performed more conveniently and indefinitely. The benefits of HoBET compared to supervised hospital-based exercise training include ease of access, convenience, and flexibility. However, the potential disadvantages of HoBET include the lack of direct supervision and the risk of suboptimal effort to attain the required level of intensity to achieve the desired benefit of exercise. Only three studies have investigated the benefit of HoBET in cirrhotic patients. These studies demonstrated increased aerobic capacity, increased muscle mass, and improved glycemic control. All three studies also showed the safety of HoBET in cirrhotic patients, including in decompensated cirrhotic patients on a waiting list for liver transplantation [12–14]. However, all three studies enrolled a small number of patients (range: 6–37). One study used a cycle ergometer as home-based exercise, which could be difficult to duplicate in a real-life setting due to the unaffordability of this machine by many patients. Another study investigated in compensated cirrhotic patients with primary aim to improve aerobic capacity and glycemic control and the last trial studied decompensated cirrhotic patients awaiting for liver transplantation with the primary aim to determine of safety of exercise. Therefore, there was limited information of the efficacy and safety of cirrhotic patients. The results of this study may be useful for both physicians and their cirrhotic patients.

The primary aim of this study was to evaluate the effect of 12-week HoBET on aerobic capacity in cirrhotic patients. The secondary aims were to evaluate the effect of HoBET on thigh muscle mass, liver and spleen stiffness, and patient QoL.

## Methods

### Patients

This randomized controlled study was conducted at the Division of Gastroenterology of the Department of Medicine, Faculty of Medicine Siriraj Hospital, Mahidol University, Bangkok, Thailand, from February 2019 to August 2020. Cirrhotic adults aged 18–65 with a Child-Turcotte-Pugh (CTP) score ≤ 6 were eligible for inclusion. Cirrhosis was diagnosed using computed tomography or magnetic resonance imaging, transient elastography with liver stiffness measurement ≥ 13 kPa, or liver biopsy (Metavir fibrosis score = 4). The etiologies of cirrhosis included chronic hepatitis B (CHB) on antiviral drugs with virological suppression, chronic hepatitis C (CHC) with the sustained virological response (SVR), alcohol with abstinence ≥ 1 year, or non-alcoholic steatohepatitis (NASH). Patients were excluded if they had one or more of the following conditions: (1) body mass index (BMI) ≥ 28 kg/m^2^; (2) large esophageal varices (EV) or uncontrolled EV or gastric varices; (3) active malignancy, including hepatocellular carcinoma; or, (4) significant comorbid diseases, such as cardiac diseases (coronary artery disease, cardiac arrhythmia, impaired ejection fraction < 60%, or positive exercise stress test ≥ 1 mm ST depression), chronic renal failure on dialysis, hematologic diseases (hemoglobin < 11 g/dL or platelet count < 50,000 cell/mm^3^), myopathy, or chronic lung diseases (severe asthma and chronic obstructive pulmonary disease).

All patients were screened by only single investigator (PS) for eligibility during visits to our outpatient liver clinic. The study protocols and potential risks associated with study interventions were discussed before informed consent was provided by all participants before participation in the study. This study was approved by the Siriraj Institutional Review Board, and was registered in the Thai Clinical Trials Registry (reg. no. TCTR20190926002).

### Study design

Patients were assigned by a computer-generated block of 4 randomizations with concealed allocation to the HoBET group or the control group for 12 weeks. Moderate-intensity continuous training (MICT) instruction was provided to the patients in the HoBET group by a sports scientist (YJ) for two weeks before the start of the study and every four weeks until the end of the study. Patients also received an instructional DVD for viewing at home. The MICT program included a 5-min warm-up, aerobic and isotonic exercise for 30 min, and a 5-min cooldown (Table 1 and Additional file 1: Supplement data 1). The exercise program was performed at least four times a week with moderate intensity to maintain the heart rate between 60 and 80% of the maximum heart rate. Heart rate and activities were recorded using a Garmin® 235 watch (Garmin International, Inc., Schaffhausen, Switzerland). A Garmin® 235 watch is a wrist-worn activity tracker utilizing green light-emitting diode optical sensors to measure the amount of light refracted in the blood vessels using photoplethysmography to calculate the heart rate in beat per minutes. There was evidence supporting the use of Garmin® 235 watch that showed accurate measurements of heart rate during rest, cycling, treadmill running, and rapid arm movement compared to the standard chest strap monitor [15, 16]. Exercise adherence was defined as ≥ 80% compliance with prescribed exercise sessions, which was assessed by diary record and Garmin watch. The heart rate was recorded for one consecutive month by the wrist-worn activity tracker, then downloaded to the computer. The correlation between the heart rate and diary record was assessed by PS. We contacted patients by telephone call every two weeks to encourage exercise program adherence and to monitor adverse events (musculoskeletal injury, fall, and hospitalization). Patients in the control group were given general exercise advice, and they were encouraged to continue their regular daily activity routine. Patients were asked to record their exercise sessions in the provided study diary and in the Garmin watch, and all patients were followed-up every four weeks at the outpatient liver clinic until the end of the study.

**Table 1:** The moderate-intensity continuous training program included a 5-min warm-up, 30-min aerobic and isotonic exercise, and 5-min cooldown

Physical exercise	Time	Repetitions per set	Sets per session
Warm up	5 min		
Squat	30 min	15	2
Leg lunge		15	2
Sumo squat		15	2
Chest press		15	2
Back extension		15	2
Shoulder press		15	2
Lateral raise press		15	2
Arm extension		15	2
Bicep curl		15	2
Trunk twist		15	2
Cool down	5 min		

Per session exercise protocol, patients in home-based exercise training group had to begin from Squat exercise until trunk twist exercise continuously as demonstrated in the table. The participants had to perform a total of 15 repetitions for each types of exercise with two sets per session

Nutritional counseling was provided to all patients by a nutritionist regarding the optimal protein and calorie intake targets (protein 1.2–1.5 g/kg/day, and calories 35–40 kcal/kg/day). On exercise days, patients in the HoBET group were asked to consume an additional soy milk supplement (250–300 kcal) to replace the calories burned. Patients in the control group who performed general exercise were also advised to drink an additional soy milk supplement on exercise days. All patients in both groups were asked to record their daily food intake in a food diary to ensure that all participants received adequate protein and caloric intake.

Only PS and YJ recognized the randomization allocation, while the rest of the co-investigators were blinded to avoid potential assessment bias.

### Study outcomes

The primary outcome was a change in aerobic capacity according to the results of a 6-min walk test (6MWT) from baseline to post-intervention between the HoBET and control groups. The secondary outcomes were a 12-week change in thigh muscle mass, HVPG using liver stiffness (LS) and spleen stiffness (SS), and patients QoL measured using the Chronic Liver Disease Questionnaire (CLDQ) compared between groups.

### Outcome measures

Patients in both groups were assessed for all outcome measures at baseline and the conclusion of the 12-week study period.

### Aerobic capacity

All patients in both groups were assessed for aerobic capacity using the 6MWT according to the American Thoracic Society guideline [17]. This objective test was measured by investigator (WB) blinded to all clinical data.

### Thigh muscle mass

**Fig. 1:**
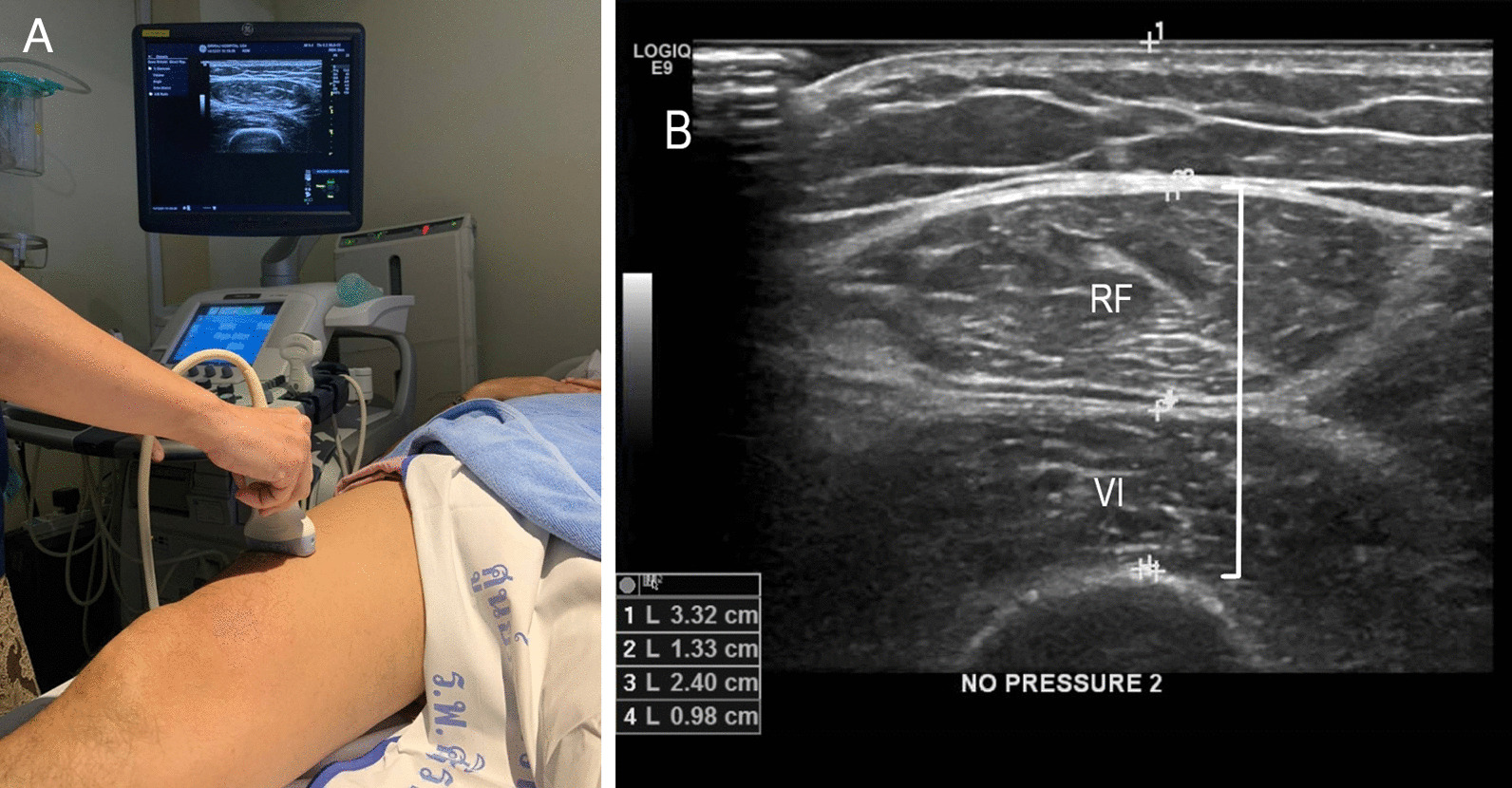
Ultrasonography was used to measure thigh muscle thickness. The locations of measurement were the depth of the right quadriceps muscle at one-third and one-half of the total distance from the top of the patella to the iliac crest (**A**). The depth of the right quadricps muscle composed of the rectus femoris and vastus intermedius (**B**). Two readings were obtained for each point: a compression reading and a feather weight reading. Abbreviation: RF, rectus femoris; VI, vastus intermedius

Thigh circumference and thigh muscle thickness (TMT) were measured [18]. Bedside ultrasound was used to measure the depth of the right quadriceps muscle (the rectus femoris and vastus intermedius). The depth of the right quadriceps muscle was measured at one-third and one-half of the total distance from the top of the patella to the iliac crest. Two readings were obtained for each point, as follows: (1) a compression reading was taken by pressing the probe downwards until no further compression of the muscle was possible, and (2) a feather weight reading was taken with the probe held without pressure on the thigh (Fig. 1). Measurements at both points (one-third and one-half distance) were averaged and corrected for stature (height squared) to yield average compression and feather weight indices. Thigh circumference was evaluated at the one-third point using a flexible tape measure. These measurements were performed by a single experienced radiologist (KM) who was blinded to all clinical data. TMT measurement by using ultrasonography has been proven to be reliable and valid for the accurate assessment of overall muscle mass [19].

### Anthropometric measurements

Bioelectrical impedance analysis (BIA) method (BC-420; Tanita Corporation, Tokyo, Japan) was used to measure fat body mass, muscle mass, lean body mass, and body weight. Waist circumference and BMI were also measured/calculated in all patients.

### Transient elastography

HVPG was evaluated using transient elastography (Fibroscan® 502 Touch; Echosens, Paris, France) with an M probe to measure liver and spleen stiffness. Transient elastography was painless, rapid, and easy to perform at the bedside. The examination was performed on a fasting (at least 4 h) patient lying flat on his/her back. The tip of the probe transducer was placed on the interconstal space at the level of the right lobe of the liver and the spleen. The median values of ten successful measurements were considered representative of liver and spleen stiffness and the values were expressed in kilopascals. Liver and spleen stiffness was measured by the technician with experience in performing transient elastography of more than 1000 patients, and blinded to clinical data. Transient elastography is a non-invasive technique to assess portal hypertension that was reported to correlate with HVPG measurement positively [20–22].

### Quality of life (QoL)

All patients completed the translated and validated Thai version of the CLDQ, which is a health-related quality of life questionnaire for liver disease patients, at baseline and at the end of the study [23, 24]. The questionnaire consists of 29 items in 6 domains, including: abdominal symptoms, fatigue, systemic symptoms, activity, emotional functions, and worry. Each domain express on a 1–7 scale, and higher scores indicate better patient QoL.

#### Laboratory tests

Blood tests to monitoring safety and to assess liver disease severity were performed at baseline and at the end of the study. Laboratory tests included complete blood count, electrolytes, creatinine, creatinine kinase, lactic acid dehydrogenase, serum albumin, bilirubin, alanine aminotransferase, aspartate aminotransferase, and coagulogram. CTP and model for end stage liver disease (MELD) were calculated to monitor liver disease severity.

#### Sample size calculation and statistical analysis

The sample size was calculated using data from a prior study on the difference in a change in 6MWT^19^ between the HoBET and the control groups being 1.042 with a standard deviation of 33.7 m. Using this data, we calculated a minimum sample size of 15 patients in each group to achieve 80% power to detect differences between groups at a 5% significance level. Allowing an attrition rate of 30%, a total sample size of 40 patients (HoBET group n = 20, and control group n = 20) was required.

All statistical analysis was performed using SPSS Statistics version 18.0 (SPSS, Inc., Chicago, IL, USA). Data were summarized using descriptive statistics. Continuous data were analyzed by *t*-test and analysis of covariance (ANCOVA), and categorical variables were analyzed by using chi-square test or Fisher's exact test. Continuous variables are given as mean plus/minus standard deviation or median and range. Categorical variables are shown as number and percentage. A *p*-value of < 0.05 was considered statistically significant.

## Results

### Baseline characteristics

A total of 40 cirrhotic patients were enrolled. Patients were randomized to the HoBET group (n = 20) or the control group (n = 20). The mean age was 56.3 ± 7.8 years, 26 patients (65%) were male, and the mean BMI was 25.2 ± 3.0 kg/m^2^. The mean percent fat mass was 26.8 ± 8.4%, and the mean muscle mass was 47.6 ± 8.1 kg. However, the mean muscle mass in the control group was significantly lower than in the HoBET group (*p* = 0.01). All patients were CTP A, and CHB and CHC were the major causes of cirrhosis (47.5% and 45%, respectively). Baseline laboratory tests were similar between groups, except for hemoglobin level, which was significantly lower in the control group (13.3 ± 1.7 g/dL and 14.6 ± 1.4 g/dL in control and HoBET, respectively; *p* = 0.01, 95% CI −2.3 to −2.9) (Table 2).

**Table 2:** Baseline characteristics compared between the home-based exercise training and control groups

Characteristics	HoBET (n = 20)	Control (n = 20)	*p-*value
Age (years), mean ± SD	55.6 ± 8.9	57.1 ± 6.7	0.56
Male gender, n (%)	15 (75%)	11 (55%)	0.18
Etiology of cirrhosis, n (%)			
Hepatitis B infection	11 (55%)	8 (40%)	0.34
Hepatitis C infection	7 (35%)	11 (55%)	0.20
Alcohol	1 (5%)	1 (5%)	1.00
Non-alcoholic steatohepatitis	1 (5%)	0 (0%)	1.00
Severity of liver disease			
MELD, mean ± SD	7.95 ± 1.5	7.95 ± 1.3	1.00
CTP score, median (range)	5 (5–5)	5 (5–5)	1.00
History of small EV, n (%)	2 (10%)	4 (20%)	0.66
History of previous EV bleeding, n (%)	1 (5%)	2 (10%)	1.00
BMI (kg/m^2^), mean ± SD	25.3 ± 2.7	25.2 ± 3.4	0.92
Waist circumference (cm), mean ± SD	91.3 ± 8.0	90 ± 7.4	0.61
%Fat, mean ± SD	24.6 ± 7.0	29.1 ± 9.1	0.09
Muscle mass (kg), mean ± SD	50.8 ± 7.6	44.4 ± 7.5	***0.01***
Laboratory results, mean ± SD			
Fasting blood sugar (mg/dL)	111.9 ± 25	106.1 ± 35.7	0.56
Cholesterol (mg/dL)	174.1 ± 22.8	172.4 ± 32.2	0.85
LDL-C (mg/dL)	101.7 ± 21.4	100.3 ± 32.8	0.88
Hb (g/dl)	14.6 ± 1.4	13.3 ± 1.7	***0.01***
Platelet count (X10^9^/L)	169 ± 80	146 ± 45	0.27
Albumin (g/dl)	4.4 ± 0.3	4.3 ± 0.4	0.85
Total bilirubin (mg/dl), median (range)	0.72 (0.6–0.9)	0.63 (0.4–0.9)	0.21
ALT (U/L), median (range)	25 (22–49)	27 (20–37)	0.53
International normalized ratio (INR)	1.1 ± 0.08	1.1 ± 0.09	0.55
Creatinine (mg/dl)	0.9 ± 0.2	1.3 ± 2.3	0.43
6-min walk test (meters), mean ± SD	479.8 ± 61.1	470.6 ± 80	0.69
Transient elastography, mean ± SD			
Liver stiffness (kPa)	14.0 ± 9.6	16.6 ± 9.2	0.39
CAP (dB/m)	235.3 ± 44.3	229.3 ± 47.9	0.69
Spleen stiffness (kPa)	25.8 ± 16.2	33.7 ± 25.9	0.64
Thigh muscle mass, mean ± SD			
Thigh circumference (cm)	49.2 ± 3.4	48.3 ± 4.7	0.49
Average feather index (cm/m^2^)	0.82 ± 0.16	0.86 ± 0.26	0.61
Average compression index (cm/m^2^)	0.62 ± 0.12	0.67 ± 0.17	0.35
Quality of life, mean ± SD			
Abdominal symptoms domain	5.9 ± 1.0	5.3 ± 1.3	0.17
Fatigue domain	5.1 ± 1.1	4.7 ± 1.1	0.29
Systemic symptoms domain	5.8 ± 0.8	5.2 ± 1.2	***0.045***
Activity domain	5.8 ± 0.8	5.3 ± 1.2	0.18
Emotional function domain	5.4 ± 1.0	4.9 ± 1.3	0.15
Worry domain	5.8 ± 1.5	5.1 ± 1.4	0.14
Average CLDQ score	5.6 ± 0.9	5.1 ± 1.1	0.43

A *p*-value < 0.05 indicates statistical significance

*ALT* alanine aminotransferase, *BMI* body mass index, *CAP* controlled attenuation parameter, *CLDQ* chronic liver disease questionnaire, *CTP* Child-Turcotte-Pugh, *EV* esophageal varices, *Hb* hemoglobin, *HoBET* home-based exercise training, *LDL-C* low-density lipoprotein cholesterol, *MELD* model for end-stage liver disease

The mean baseline 6MWT was 475.2 ± 70.4 m. The mean LS and SS measurements were 15.3 ± 9.4 kPa and 29.8 ± 21.7 kPa, respectively. The average feather index and average compression index, both of which comprised the TMT, were 0.84 ± 0.2 cm/m^2^ and 0.64 ± 0.2 cm/m^2^, respectively. The mean TMT and thigh circumference were not significantly different between groups. QoL evaluated in 6 domains was similar between groups, except that systemic symptoms were significantly higher in HoBET patients than in control group patients (*p* = 0.045, 95% CI −6.6 to −0.7) (Table 2).

### Aerobic capacity

**Fig. 2:**
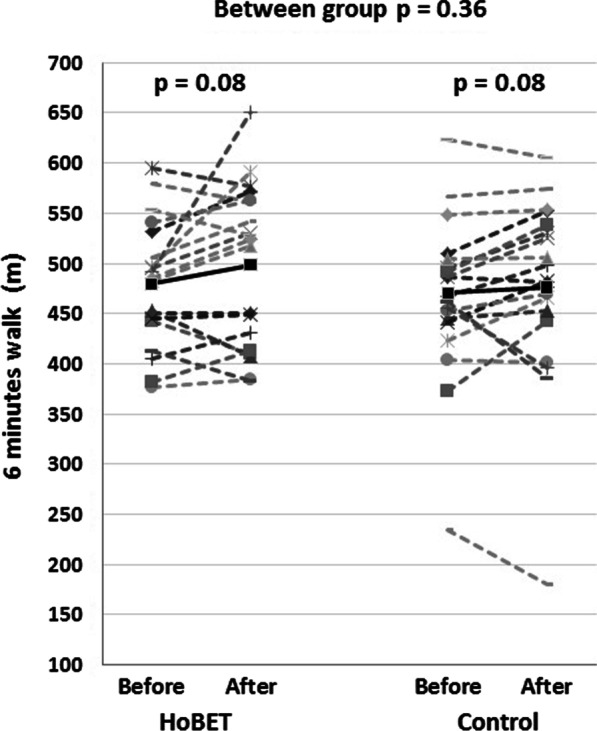
Individual patient 6-min walk test result compared between before and after the intervention for both the home-based exercise training (HoBET) group and the control group. The dotted lines represent individual patients, and the black solid lines indicate the mean before and after the intervention

Among patients in the HoBET group, the 6MWT distance tends to increase from 479.8 ± 61.1 m at baseline to 498.6 ± 77.8 m at the end of the study (*p* = 0.08, 95% CI −40.8 to 3.7). However, the difference didn't reach statistical significance. Similarly, the 6MWT distance in the control group minimally increased from 470.6 ± 80 m at baseline to 476.2 ± 91.2 m at the end of the study (*p* = 0.08, 95% CI −24.7 to 13.5). No Difference in mean 6MWT distance at the end of the study was observed between groups (Fig. 2).

### Body mass index (BMI) and anthropometric measurements

No significant change in BMI between baseline and the end of the study was observed in either group. The mean BMI of all patients remained stable at approximately 25 kg/m^2^, and there was no significant difference in mean BMI between groups. Percentage of fat and muscle mass were measured by BIA, and patients demonstrated unchanged total percent fat and muscle mass both within and between groups. (Table 3).

**Table 3:** Baseline and end of study measurements compared between home-based exercise training and control groups

Characteristics	HoBET group (n = 20)			Control group (n = 20)			Change in mean (95% CI)	*p*-value between groups* , 95%CI
	Baseline (mean ± SD)	End of study (mean ± SD)	Within group *p*	Baseline (mean ± SD)	End of study (mean ± SD)	Within group *p*		
Body mass index (kg/m^2^)	25.2 ± 2.7	25.2 ± 2.7	0.71	25.2 ± 3.4	25.0 ± 3.3	1.00	2.3 (−0.2, 0.6)	0.30 (−0.2–0.7)
% Fat	24.6 ± 7.0	25.1 ± 6.6	0.44	29.1 ± 9.1	28.3 ± 9.1	0.08	1.24 (−0.27, 2.74)	0.22 (−0.6–2.5)
Muscle mass (kg)	50.8 ± 7.6	50.6 ± 7.9	0.48	44.4 ± 7.5	44.5 ± 7.4	0.86	−0.31 (−1.24, 0.62)	0.53 (−0.7–1.3)
6MWT (meters)	479.8 ± 61.1	498.6 ± 77.8	0.08	470.6 ± 8.0	476.2 ± 91.2	0.08	13.3(−14.9–41.5)	0.36 (−15.5–41.7)
**Transient elastography**								
Liver stiffness (kPa)	14.0 ± 9.6	12.2 ± 7.6	***0.016***	16.6 ± 9.2	14.0 ± 8.1	***0.016***	0.7 (−1.2, 2.7)	0.77 (−1.3–1.8)
Spleen stiffness (kPa)	25.8 ± 16.2	36.1 ± 43.6	0.30	33.7 ± 25.9	31.7 ± 22.4	0.30	12.3 (−8.7, 33.3)	0.37 (−11.6–30.4)
**Thigh muscle mass**								
Thigh circumference (cm)	49.2 ± 3.4	49.5 ± 3.3	0.26	48.3 ± 4.7	48.2 ± 4.3	0.58	0.5 (−0.31, 1.31)	0.13 (−0.2–1.4)
Average feather index (cm/m^2^)	0.82 ± 0.16	0.89 ± 0.21	0.20	0.86 ± 0.26	0.85 ± 0.20	0.61	0.07 (−0.03, 0.16)	0.18 (−0.03–0.15)
Average compression index (cm/m^2^)	0.62 ± 0.12	0.70 ± 0.17	0.09	0.67 ± 0.17	0.63 ± 0.14	0.07	0.02 (0.02, 0.19)	***0.03*** ***(0.01–0.17)***
**Quality of life**								
Abdominal symptoms	5.9 ± 1.0	5.9 ± 1.3	0.95	5.3 ± 1.3	5.3 ± 1.4	1.0	0.01 (0.7, 0.7)	0.62 (−0.5–0.8)
Fatigue	5.1 ± 1.1	5.6 ± 0.9	***0.009***	4.7 ± 1.1	4.9 ± 1.1	***0.02***	0.3 (−0.1, 0.6)	***0.05*** ***(0.0–0.67)***
Systemic symptoms	5.8 ± 0.8	6.0 ± 0.7	0.22	5.2 ± 1.2	4.7 ± 0.8	0.95	0.2 (−0.3, 0.6)	0.12 (−0.09–0.8)
Activity	5.8 ± 0.8	6.3 ± 0.9	***0.01***	5.3 ± 1.2	6.3 ± 1.0	0.13	0.3 (−0.3, 0.9)	0.08 (−0.06–0.97)
Emotional function	5.4 ± 1.0	5.8 ± 0.9	***0.03***	4.9 ± 1.3	5.8 ± 0.9	***0.03***	−0.05 (−0.5, 0.4)	0.68 (−0.3–0.5)
Worry	5.8 ± 1.5	5.9 ± 1.1	0.66	5.1 ± 1.4	5.4 ± 1.4	0.06	−0.13 (−0.7, 0.5)	0.72 (−0.4–0.6)
**Average CLDQ**	5.6 ± 0.9	5.9 ± 0.8	***0.03***	5.1 ± 1.1	5.3 ± 1.1	***0.01***	0.09 (−0.2, 0.4)	0.23 (−0.7–2.8)

A *p*-value < 0.05 indicates statistical significance

*p-value calculated by analysis of covariance (ANCOVA)

*6MWT* six-minute walk test, *CLDQ* chronic liver disease questionnaire, *HoBET* home-based exercise training

### Thigh muscle mass

The average compression index non-significantly increased from 0.62 cm/m^2^ at baseline to 0.70 cm/m^2^ at the end of the study in the HoBET group, and it non-significantly decreased from 0.67 to 0.63 cm/m^2^ in the control group. However, the difference between groups at the end of the study was statistically significant at a *p*-value of 0.03, (95% CI 0.01 to 0.17). The average feature index and thigh circumference remained unchanged in the HoBET group. However, a minimal decrease in those two parameters was observed in the control group. In summary, thigh muscle mass indices and thigh circumference were no statistical differences between HoBET and the control group. (Table 3).

### Transient elastography

Transient elastography was performed to evaluate LS and SS. LS decreased from 14 to 12.2 kPa in the HoBET group (*p* = 0.016, 95% CI 1.1 to 4.0), and decreased from 16.6 to 14 kPa in the control group (*p* = 0.016, 95% CI 0.38 to 3.2) after 12 weeks. However, there was no difference observed between groups. SS was also performed to evaluate portal hypertension. SS non-significantly increased from 25.8 to 36.1 kPa in the HoBET group, and it non-significantly decreased from 33.7 to 31.7 kPa in the control group. Again, there was no statistical differences between groups (Table 3).

### Quality of life (QoL)

The average score for health-related QoL was not significantly different between groups at the end of the study, except for the fatigue domain, which was significantly improved in the HoBET group compared to the control group (*p* = 0.05, 95% CI 0.0–0.67). The activity domain was significantly improved in the HoBET group (*p* = 0.01, 95% CI −1.0 to −0.2), but not in the control group (*p* = 0.13, 95% CI −0.7 to 0.1). There was no statistical difference between the two groups ragarding the activity domain at the end of the study (*p* = 0.08, 95% CI −0.06 to 0.97). The emotional function scale was significantly increased in both the HoBET (*p* = 0.03, 95% CI −0.8 to −0.04) and control groups (*p* = 0.004, 95% CI −0.76 to −0.2); however, there was unchanged between groups at the end of the study. Other domains, including systemic symptoms, abdominal symptoms, and worry, showed no statistical difference within or between groups. (Table 3).

### Adherence to exercise in HoBET group

The major concern in the HoBET group was the ability of participants to achieve a good compliance defined by at least 80% of an actual execise performed during study being moderate-intensity, given the target heart rate between 60 and 80% of the maximum heart rate and being regulary performed with a frequency of at least 4 times a week. In the HoBET group, 15 of 20 patients (75%) achieved good compliance. There was no droput in this study.

### Adverse events

No patients in this study experienced liver deterioration during the study period. There was no portal hypertension-related complication, such as variceal bleeding or new-onset ascites, at any time during the study, even though one patient in the HoBET group had a history of variceal bleeding before enrollment. There were also no reports of intervention-related injuries in either group during the study.

## Discussion

Many current studies have shown the advantages of exercise in cirrhotic patients. Nevertheless, the clinical practice guidelines for patients with chronic liver disease, including cirrhosis, do not recommend exercise for these patients, except for patients with NASH, which showed benefit from exercise. One major exercise-related concern in cirrhotic patients is increased portal hypertension with variceal bleeding. Although much evidence has demonstrated the benefit of exercise in patients with chronic liver disease, including improved aerobic capacity, increased muscle mass, improved QoL, and decreased portal hypertension, most studies were conducted using supervised exercise in hospitals. However, evidence specific to the benefit and safety of home-based exercise is comparatively scarce. The purpose of this study was to investigate the benefit and safety of home-based exercise in cirrhotic patients.

All patients enrolled in this study had compensated cirrhosis with well-controlled underlying liver diseases, and all had a CTP score of 5. Notably, our patients received specific treatment before study enrollment, such as CHB patients who received antiviral therapy, CHC patients with SVR, and abstinence of alcohol in patients with alcoholic liver disease. Only well-controlled compensated cirrhotic patients were enrolled in this study because we were concerned about the safety of these patients exercising unsupervised at home. Chronic viral hepatitis was the most common etiology of cirrhosis in these participants because chronic viral hepatitis is still the most common cause of cirrhosis in Thailand. Only one patient in this study had cirrhosis caused by NASH.

Several studies have demonstrated an independent association between aerobic capacity and decreased morbidity and mortality in patients awaiting liver transplantation [25, 26]. Aerobic capacity was a factor that improved as a result of an exercise in cirrhotic patients, as shown in many studies. Peak oxygen uptake (VO_2_) and 6MWT were standard parameters used to monitor aerobic capacity. Previous studies demonstrated increased peak VO_2_ and 6MWT distance after supervised in-hospital or home-based exercise for 12–16 weeks [6, 7, 13]. The 6MWT is a standardized test of functional exercise capacity, which is mainly used in patients with cardiopulmonary diseases. Several studies have reported the minimal clinically important difference (MCID) for the 6MWT. A threshold of 30.5 to 54 m has been widely accepted as representative of clinically significant change [27, 28]. However, there was no currently available data for patients with other diseases. The present study did not find a significant influence of exercise on 6MWT distance at the end of the study; furthermore, the changes in 6MWT distance in both groups did not exceed MCID level. A possible explanation is that we used only 6MWT to measure aerobic capacity. The six-minute walk test was not a sufficiently sensitive method to detect cirrhotic patients who had baseline 6MWT above 250 m [7]. Since all patients in our study were compensated cirrhosis and most patients expressed high motivation to exercise, 6MWT alone may not sufficiently illustrate the benefit of exercise on aerobic capacity.

Muscle mass was shown to improve after exercise in both hospital-supervised and home-based exercise [6–9, 13]. Loss of muscle mass was correlated with sarcopenia, which is a predictor of liver-related mortality in cirrhotic patients [29, 30]. TMT was correlated with muscle mass at the third lumbar vertebrae, and it can be performed bedside. In the present study, TMT and thigh circumference were lower than the values reported from previous studies. The likely explanation is that all patients in our study were Asian and had a lower BMI; however, most patients in previous studies were Caucasian and had a higher BMI [7, 13]. Notably, patients in the HoBET group had increased average feather index, average compression index, and thigh circumference at the end of the study. In contrast, patients in the control group had a mean decrease in all three major parameters at the end of the study. However, there was no significant change in thigh muscle mass. The possible explanation was the lower intensity of the exercise, which was a moderate intensity in this study compared with high intensity in other studies. Nevertheless, our study demonstrated that home-based exercise is particularly safe at least in moderate intensity. A future study investigating the benefit of higher intensity of exercise in a home-based setting with concern regarding increased risk of an adverse event is warranted.

Many exercise studies have evaluated QoL before and after an exercise-related intervention, and all of them reported improvement in QoL as assessed by CLDQ or SF-6 [7, 11, 13]. Our study used only the CLDQ to evaluate 6 domains. Although the mean total CLDQ score was not significantly different between pre- and post-study, the fatigue and activity domains both showed significant improvement at the end of the study. Fatigue was improved both within and between groups, whereas the activity domain revealed only significant within group improvement. Fatigue is common in cirrhotic patients and is difficult to treat with medication; however, exercise was shown to relieve fatigue in cirrhotic patients [31]. Our study showed the benefit of a 12-week HoBET program for improving cirrhotic patients' QoL by reducing fatigue symptoms.

Data specific to the effect of exercise on portal hypertension is limited. Only two studies evaluated the benefit of supervised exercise on the degree of portal hypertension using HVPG measurement. One trial was a small study that enrolled only 25 patients. That study found a decreased HVPG and improved nutritional status without a change in body weight [10]. Another study examined patients with NASH and coexisting cirrhosis [11]. All patients were enrolled in a single-arm to control weight and diet, and all participants had lost weight by the end of the study. The level of HVPG decreases correlated with the level of weight reduction. Therefore, the effect of exercise on HVPG may involve weight loss in patients with NASH and cirrhosis. In our study, LS and SS were used to indirectly determine portal pressure instead of conventional HVPG measurement. LS and SS were reported to have a good correlation with HVPG measurement for evaluation of portal pressure. The results of the present study showed that patients in both the HoBET and control groups had significantly decreased LS level at the end of the study within the group, but no significant difference between groups was observed. Improvement in LS and SS as measured by elastography might be related to regression of hepatitis activity in our patients because they had a well-controlled disease [32, 33]. Our study did not show any advantage of exercise on portal hypertension. This could be due to insufficient statistical power due to our small sample size, or our method of measuring portal pressure may have lacked sufficient sensitivity to detect a difference between groups.

No adverse events, such as gastrointestinal bleeding, new ascites formation, or musculoskeletal injury, were found or reported in either group during the 12-week duration of the study. However, we enrolled only compensated cirrhotic patients, and only a few of those had a history of variceal bleeding (and 15% of those had small varices). These results indicate the safety of our HoBET program among patients with compensated liver cirrhosis.

### Strengths and limitations

This study has several strengths. First, the exercise program in this study was simple, easy to perform, and required no expensive equipment. Moreover, these exercises can be performed in a normal residence and without the need for a large space. Second, all patients in this study had exercise activity data monitored and recorded using a smartwatch. Third, all parameters for evaluating the effect of exercise in cirrhotic patients were measured, including aerobic capacity, muscle mass, portal hypertension, and QoL.

This study also has some limitations. First, the study enrolled only very well compensated cirrhotic patients. We did not enroll cirrhotic CTP B or C patients due to concerns about patient safety while performing home-based exercises. Second, most of our patients in the control group exercised regularly, either due to our pre-study advice or because that is what they normally do. Therefore, control group results may not accurately reflect the inactive state that is common among many cirrhotic patients. This factor could also have confounded the comparisons of differences between groups. Third, there were a total of 40 patients enrolled in this study. Future studies with a larger sample size may demonstrate the benefit of moderate-intensity exercise on these outcomes. Finally, changes in exercise capacity, muscle mass, and portal hypertension were assessed using simple methods used in clinical practice. More sophisticated methods of evaluation and measurement could have yielded different results. That acknowledged, the benefit of the measurement methods that we used is that they are affordable, widely available, used in routine clinical practice, and they do not require specific expertise. However, future study should employ more sensitive and accurate techniques, such as cardiopulmonary exercise test (peak VO_2_), CT for muscle mass assessment, and HVPG measurement for assessing portal hypertension, to confirm the results of this study.

## Conclusions

A 12-week home-based exercise training program was found to be safe and practical for compensated cirrhotic patients. A moderate-intensity home-based exercise training program had significantly improved the quality of life in the fatigue domain. However, no significant difference was observed regarding a 6-min walk test, thigh muscle mass, or hepatic venous pressure gradient. The benefit of exercise may be demonstrated in future studies with larger sample sizes or in the setting of higher exercise intensity. The advantages of home-based exercise in cirrhotic patients require further investigation in many aspects, including the benefit of improved aerobic capacity or decreased hepatic venous pressure gradient, the long-term effects, and clinical benefit and risk in decompensated patients.

## Supplementary Information

**Additional file 1**. Moderate-intensity continuous training program in this study included ten types of exercise that were aerobic and isotonic exercise.

## Data Availability

The datasets generated during and analyzed during the current study are not publicly available due to our University’s institutional review boards (IRB) and the health insurance portability and accountability act (HIPAA) privacy rule but are available from the corresponding author on reasonable request.

## Abbreviations

QoL: Quality of life
HVPG: Hepatic venous pressure gradient
HoBET: Home-based exercise training
CTP: Child-Turcotte-Pugh
CHB: Chronic hepatitis B
CHC: Chronic hepatitis C
SVR: Sustained virological response
NASH: Non-alcoholic steatohepatitis
BMI: Body mass index
EV: Esophageal varices
MICT: Moderate-intensity continuous training
6MWT: 6-Minute walk test
LS: Liver stiffness
SS: Spleen stiffness
CLDQ: Chronic Liver Disease Questionnaire
TMT: Thigh muscle thickness
BIA: Bioelectrical impedance analysis
MELD: Model for end stage liver disease
VO2: Oxygen uptake
MCID: Minimal clinically important difference
