# Supplementary material for: Effect of a 12-week home-based exercise training program on aerobic capacity, muscle mass, liver and spleen stiffness, and quality of life in cirrhotic patients: a randomized controlled clinical trial
Source: BMC Gastroenterol. 2022 Feb 14;22:66. doi: 10.1186/s12876-022-02147-7 (PMC8845268; doi:10.1186/s12876-022-02147-7)
Supplement: Supplementary file 1 — Additional file 1. Moderate-intensity continuous training program in this study included ten types of exercise that were aerobic and isotonic exercise. [file 12876_2022_2147_MOESM1_ESM.doc]

**Supporting information**

**Moderate-Intensity Continuous Training (MICT)**

The MICT program includes a 5-minute warm up, aerobic and isotonic exercise for 30 minutes, and a 5-minute cool down. Perform 15 repetitions of each of the following exercises in order, moving from one to the next step. After finish all types of exercise then repeat another set. Maintain 60-80% of maximum heart rate. Continue to cycle through the exercise circuit for 30 minutes, and then cool down for 5 minutes.

**1. Squat**

**
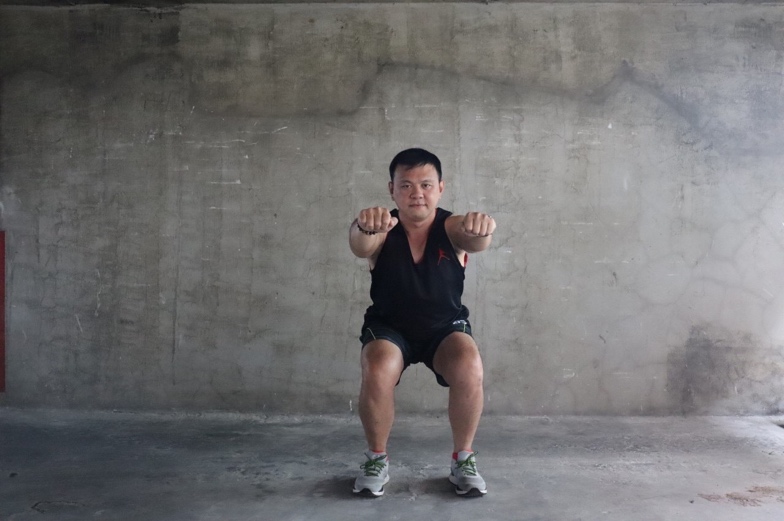

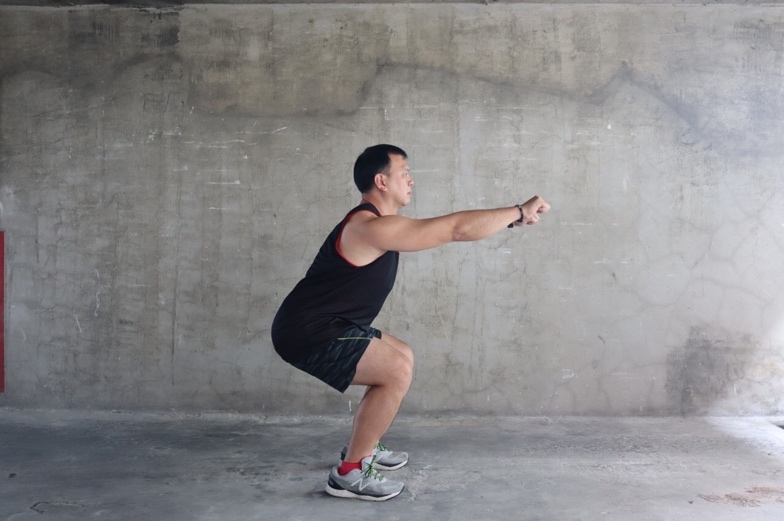
**

**2. Leg Lunge**

**
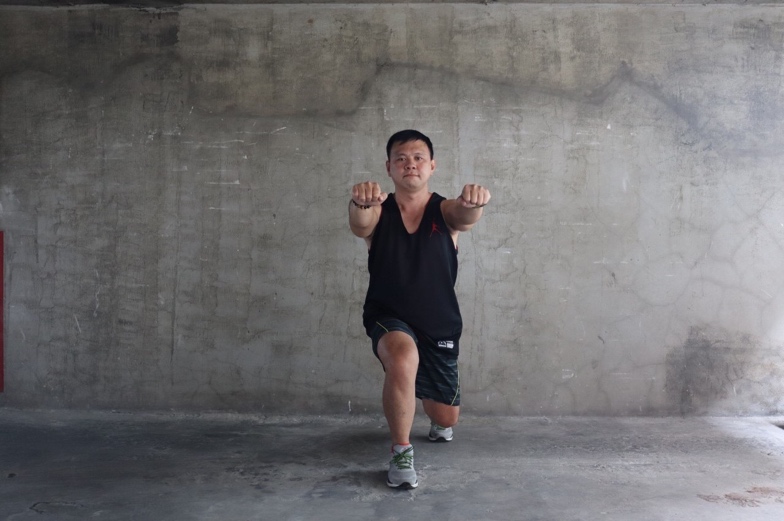

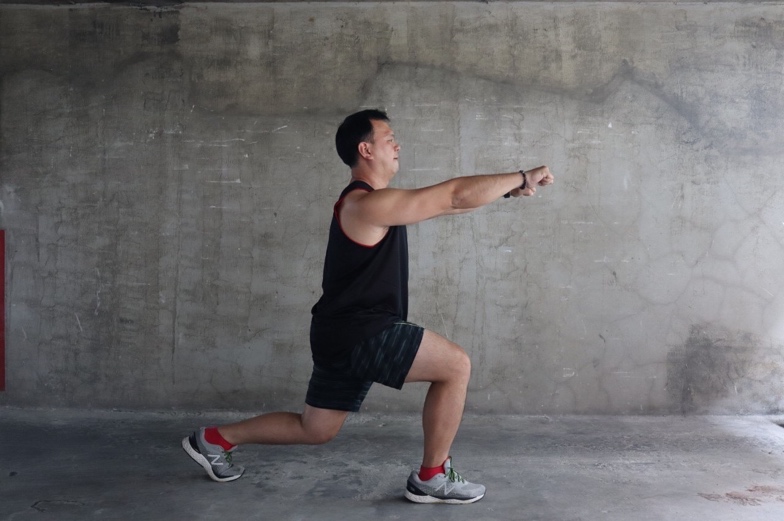
**


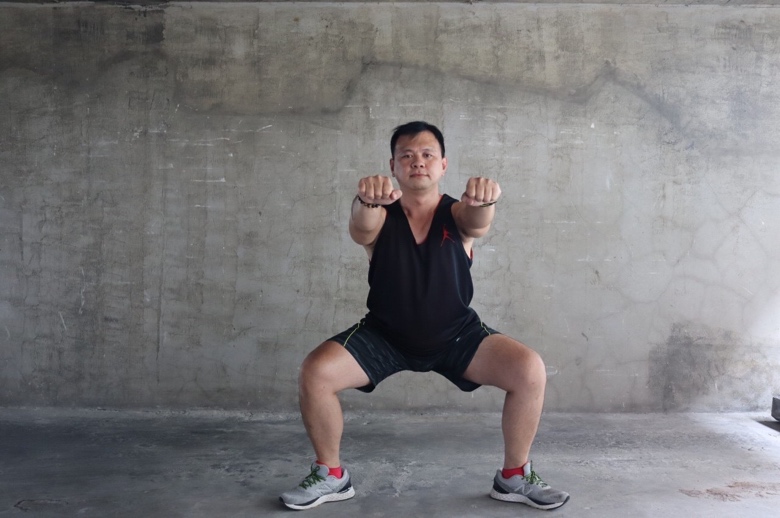

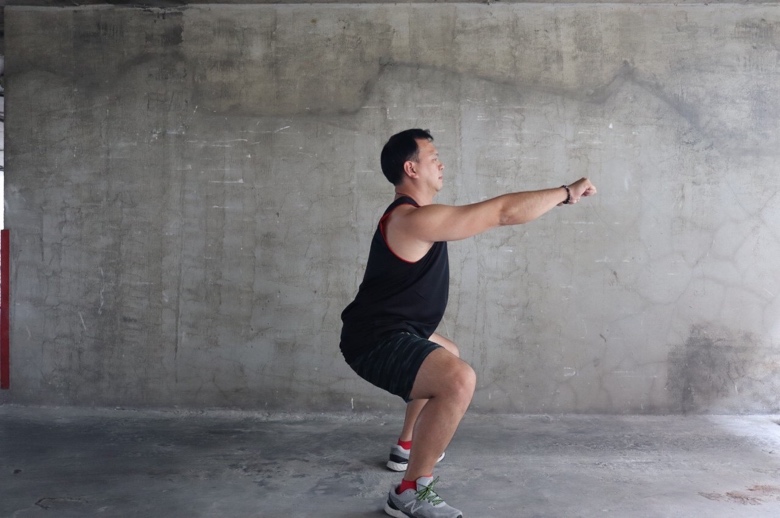
**3. Sumo Squat**


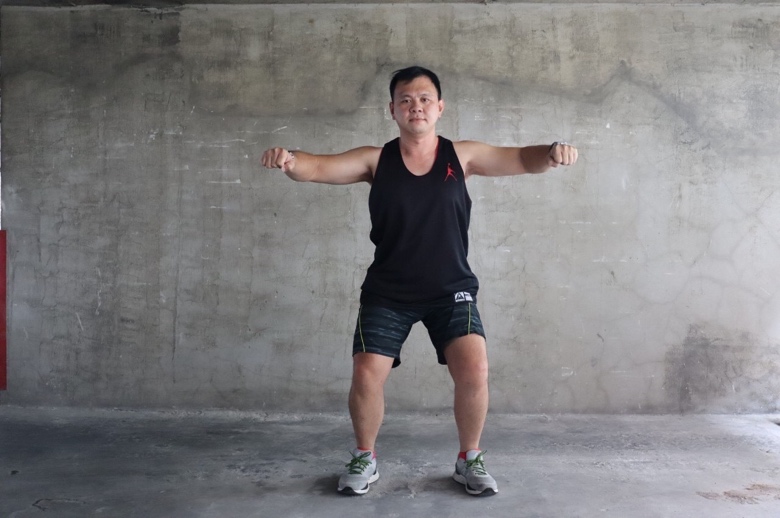

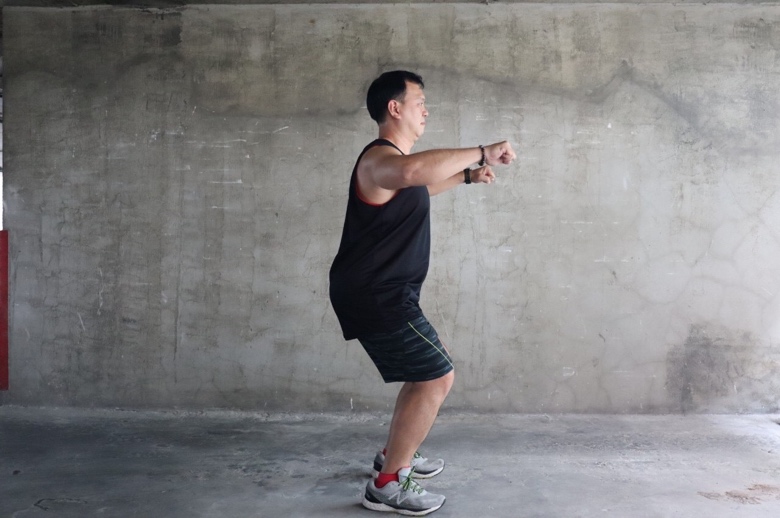

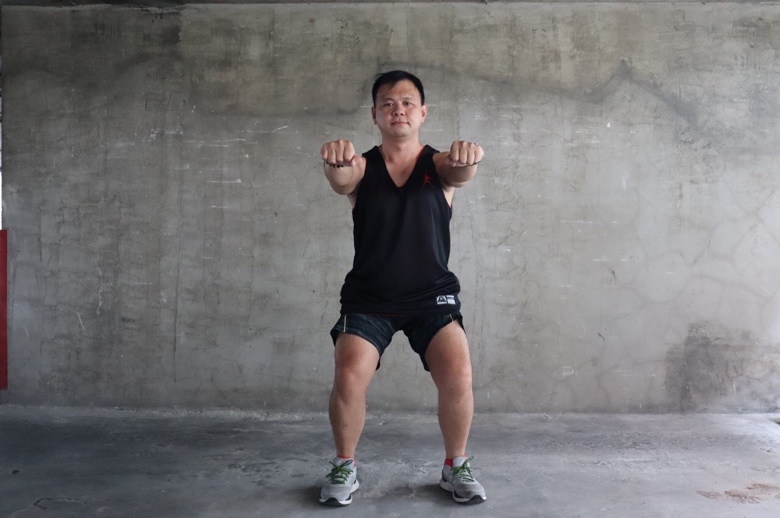

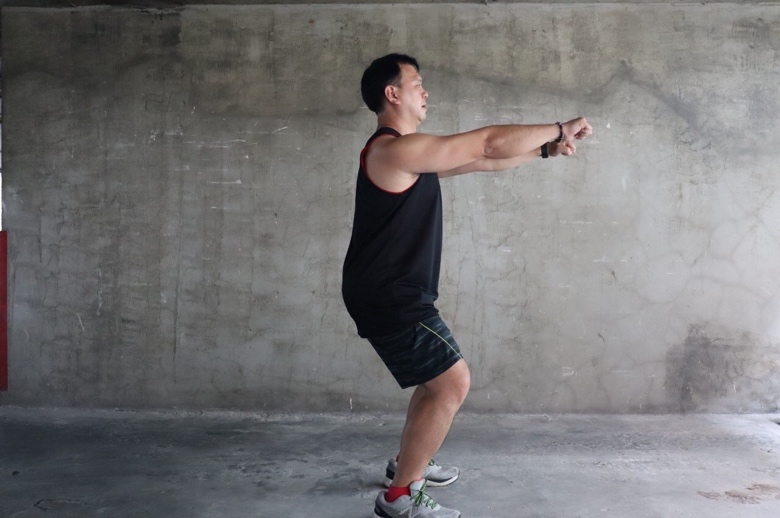
**4. Chest Press**


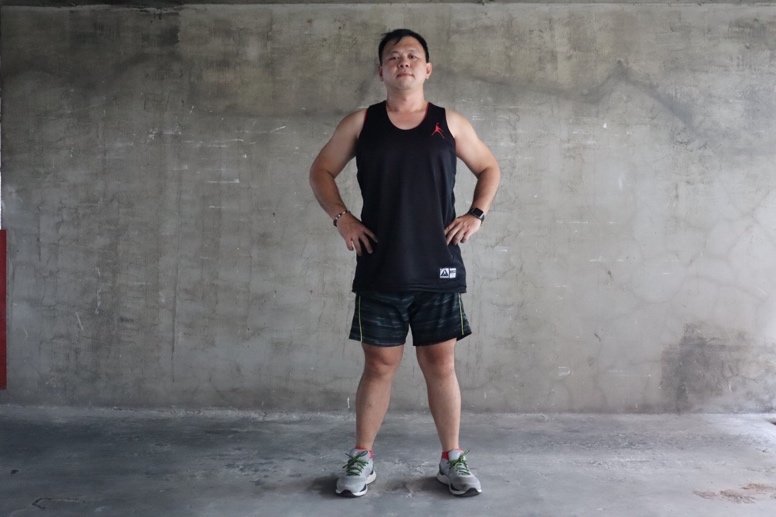

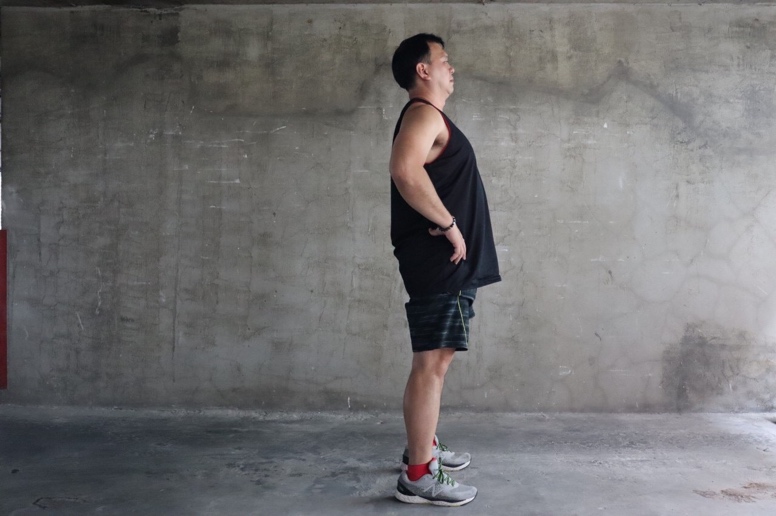

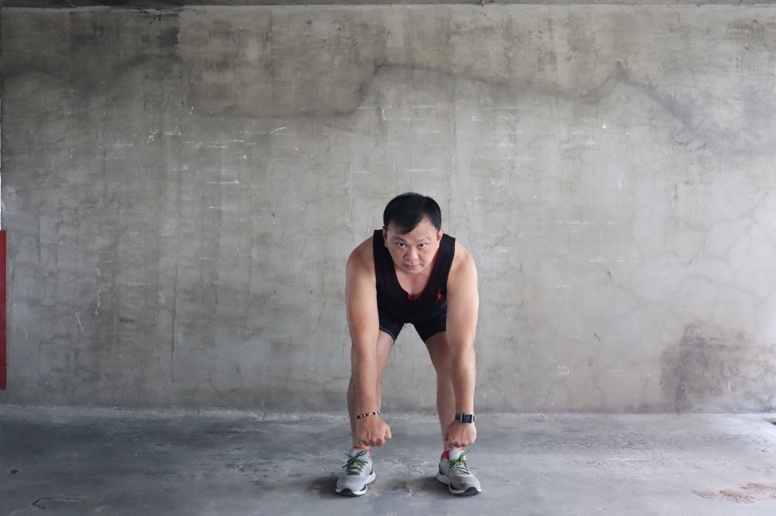

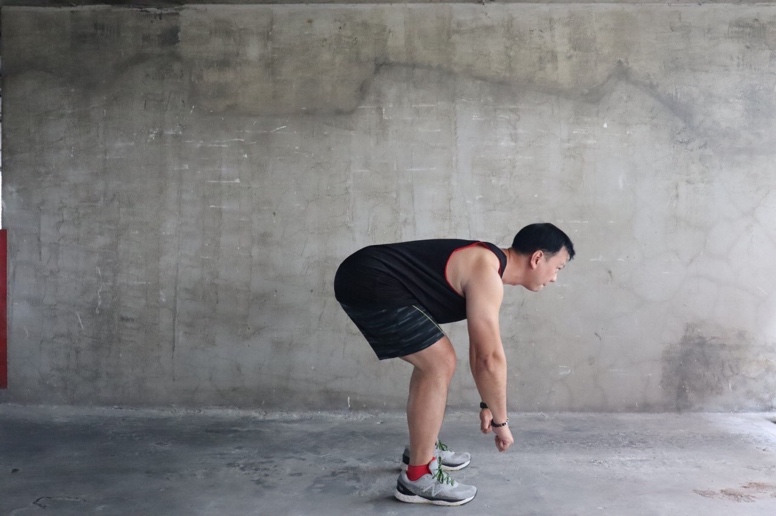
**5. Back Extension**

**6. Shoulder Press**

**
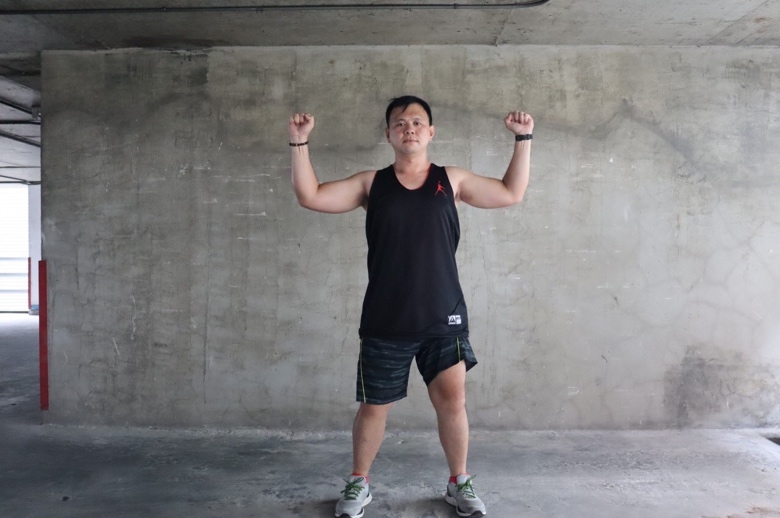

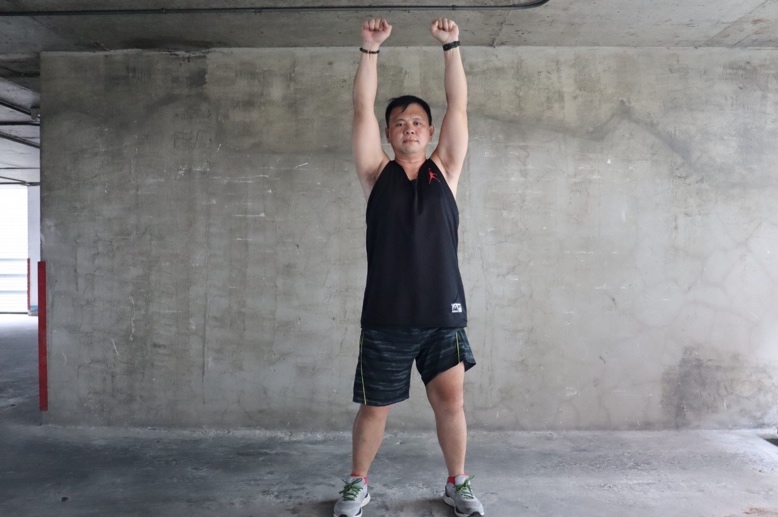
**


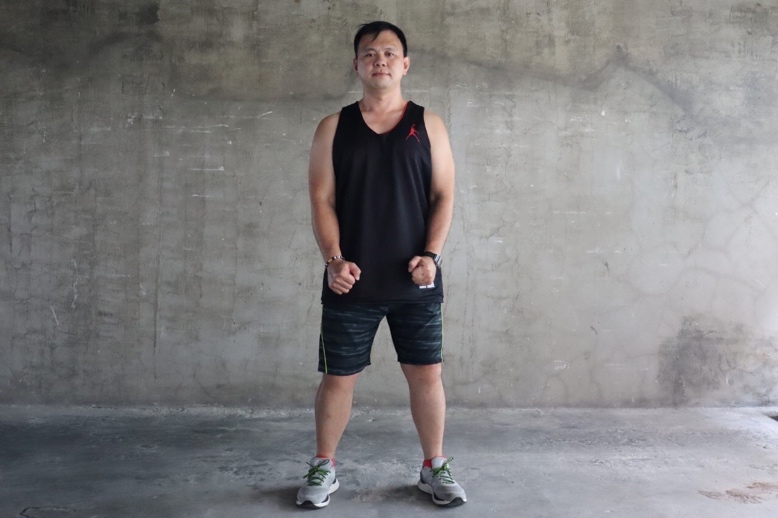

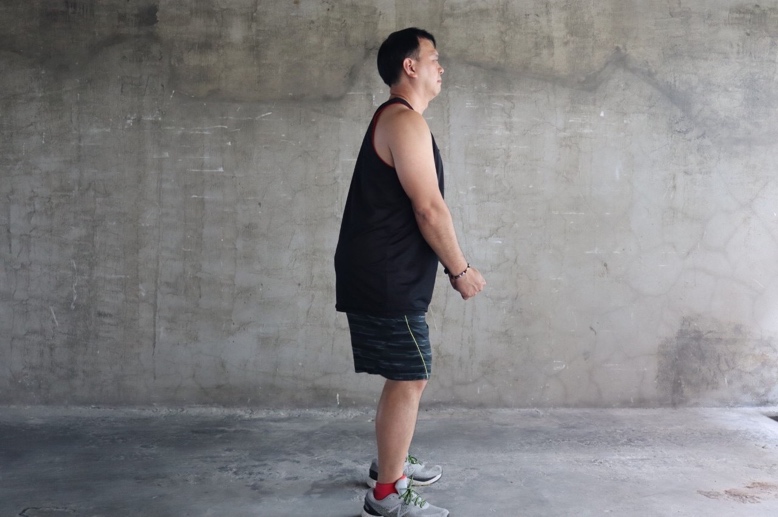

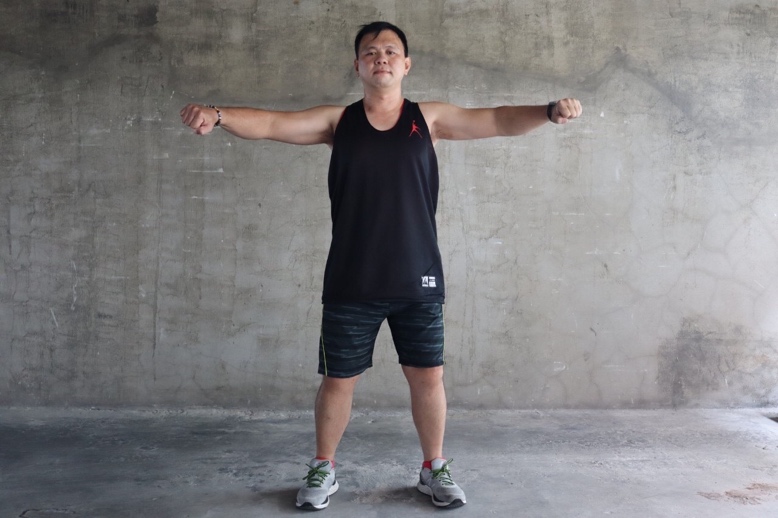

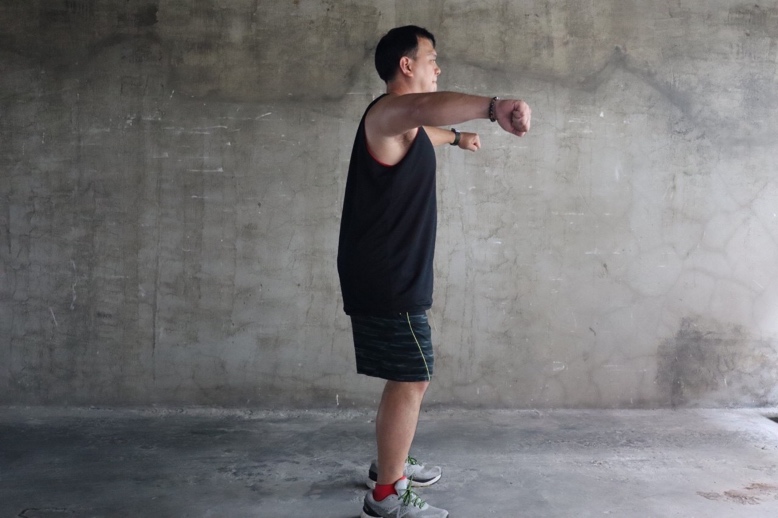
**7. Lateral Raise Press**


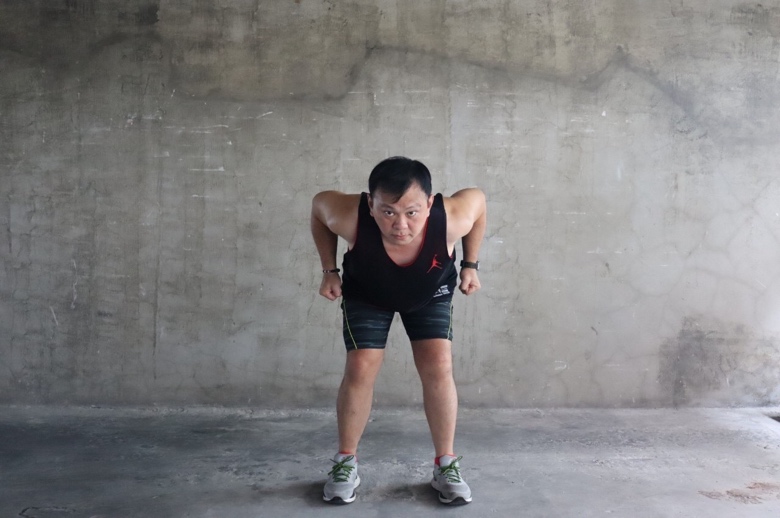

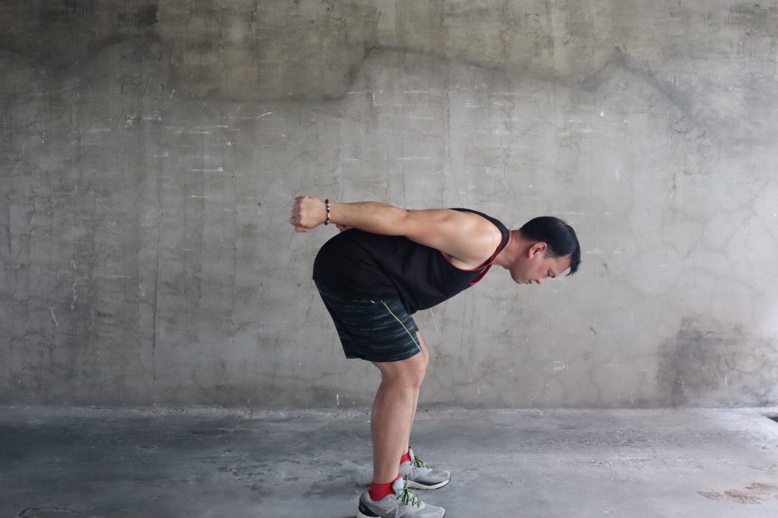
**8. Arm Extension**


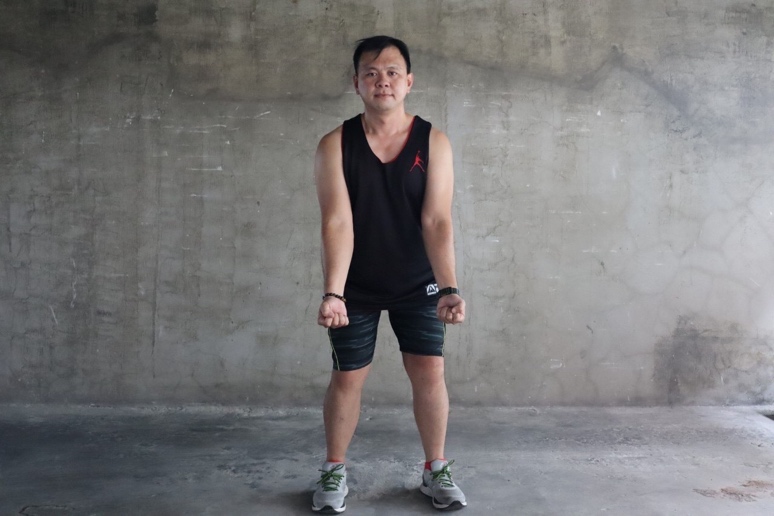

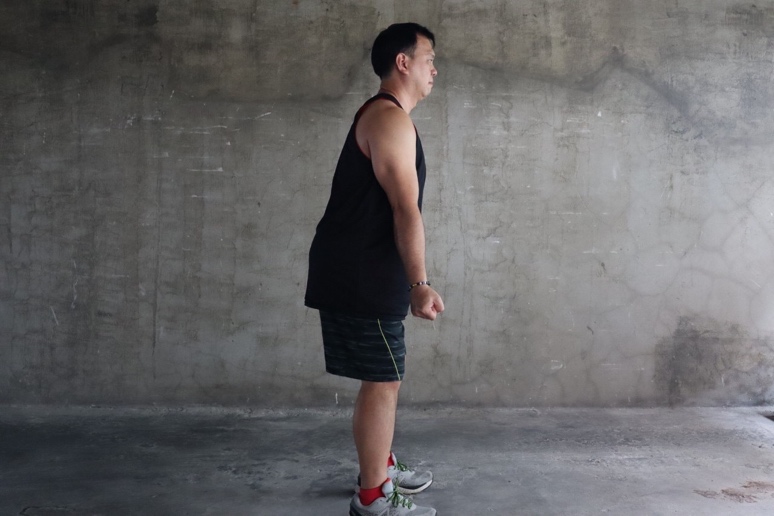

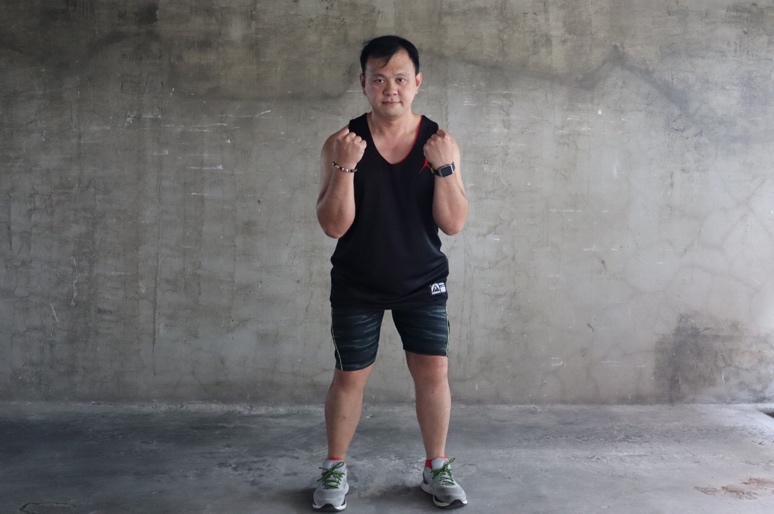

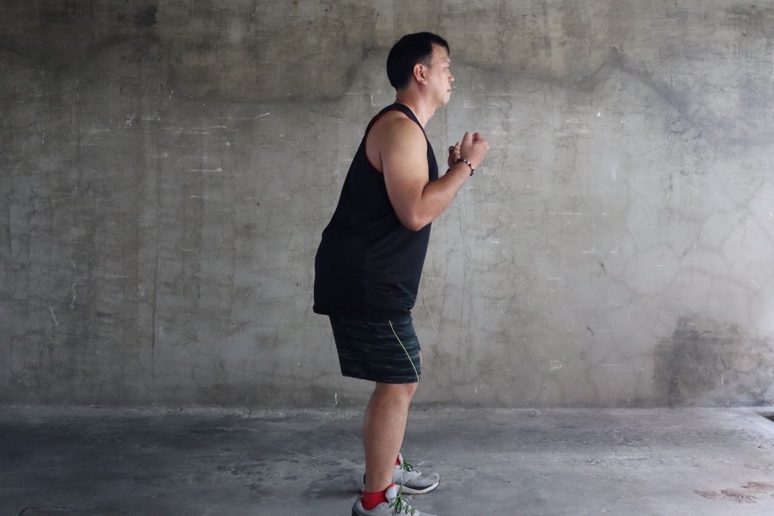
**9. Biceps Curl**


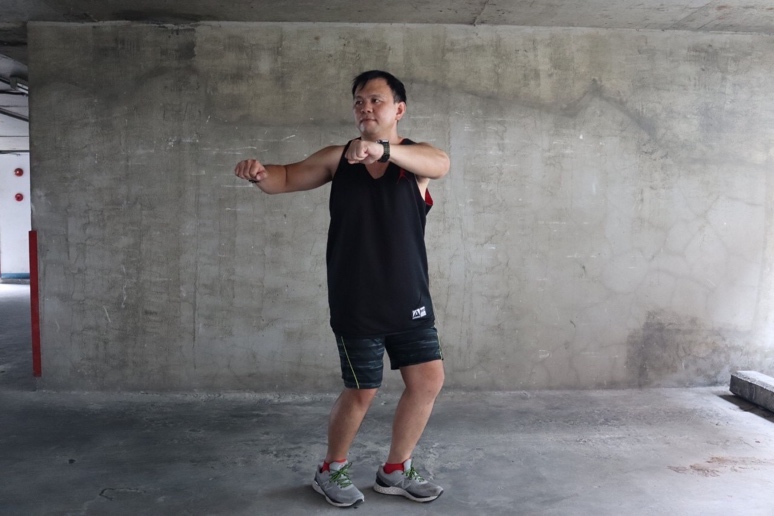

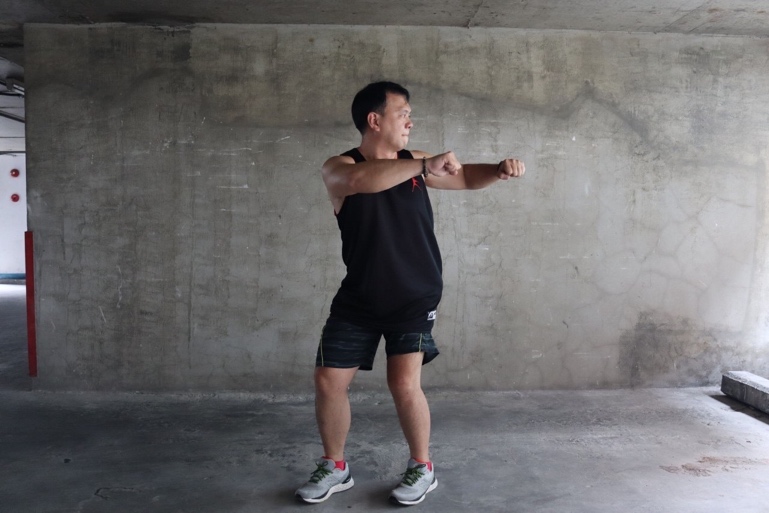
**10. Trunk Twist**
